# Supplementary material for: An aperiodic chiral tiling by topological molecular self-assembly
Source: Nat Commun. 2025 Jan 2;16:83. doi: 10.1038/s41467-024-55405-5 (PMC11696205; doi:10.1038/s41467-024-55405-5)
Supplement: Supplementary file 1 — Supplementary Information [file 41467_2024_55405_MOESM1_ESM.pdf]

Supplementary Information

An aperiodic chiral tiling by topological molecular self-assembly

Jan Voigt, Miloš Baljozović, Kévin Martin, Christian Wäckerlin, Narcis Avarvari* &

Karl-Heinz Ernst*

*Corresponding authors. Email: karl-heinz.ernst@empa.ch, narcis.avarvari@univ-angers.fr

Contents:

Supplementary Figs. 1-16

Supplementary Tables 1-2

Supplementary Equation 1

Supplementary Figures

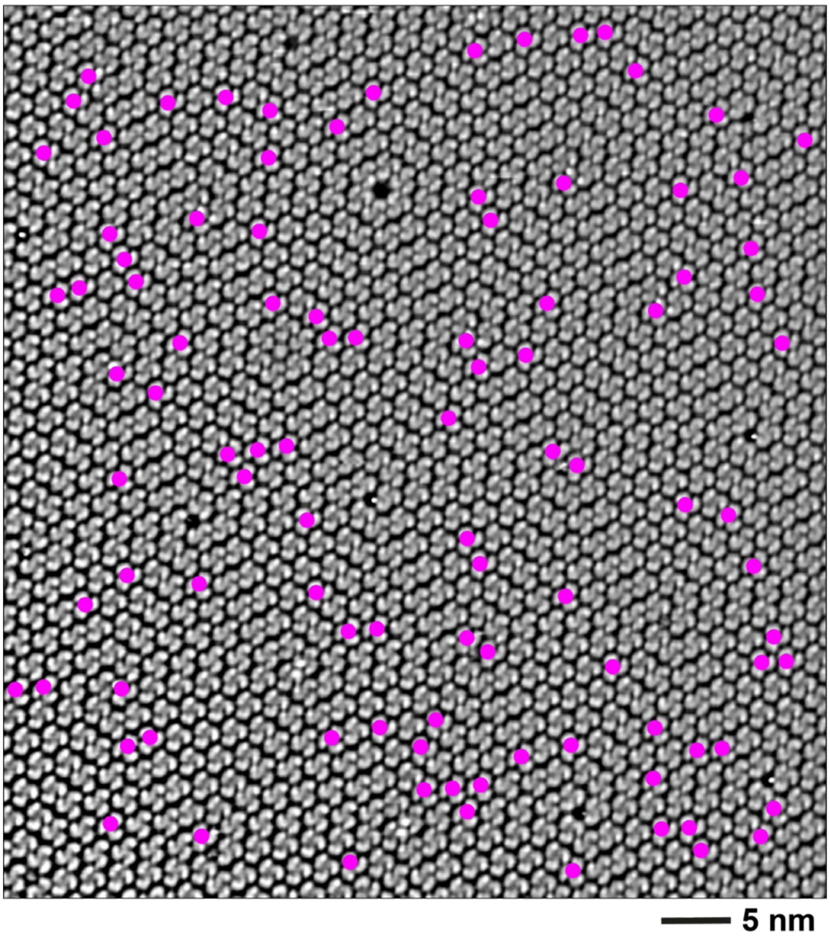

Supplementary Fig. 1 | Solid solution of enantiomers. *(P)*-enantiomers are marked with filled cyan circles for this domain with *(M)*-enantiomers as majority. The shown area is identical to the area shown in Fig. 1b.

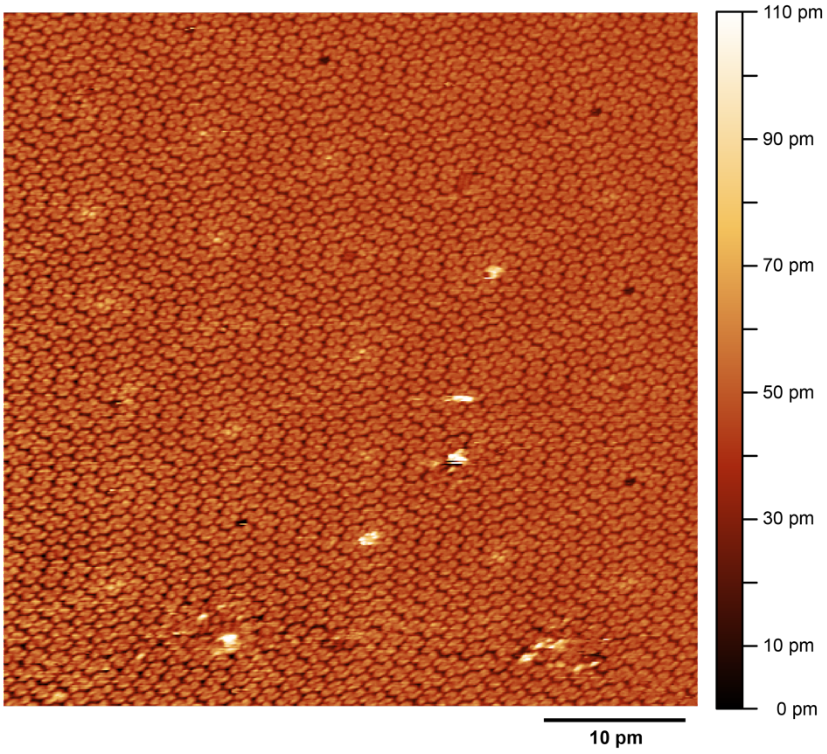

Supplementary Fig. 2 | Mirror domain assembly. STM image (without drift correction) of an area showing the opposite 'mirror world' assembly with respect to Fig.1b. The image has been taken from the same preparation but hundreds of μm away from the area shown in Fig. 1b. The color scale gives the apparent height width probed of the monolayer.

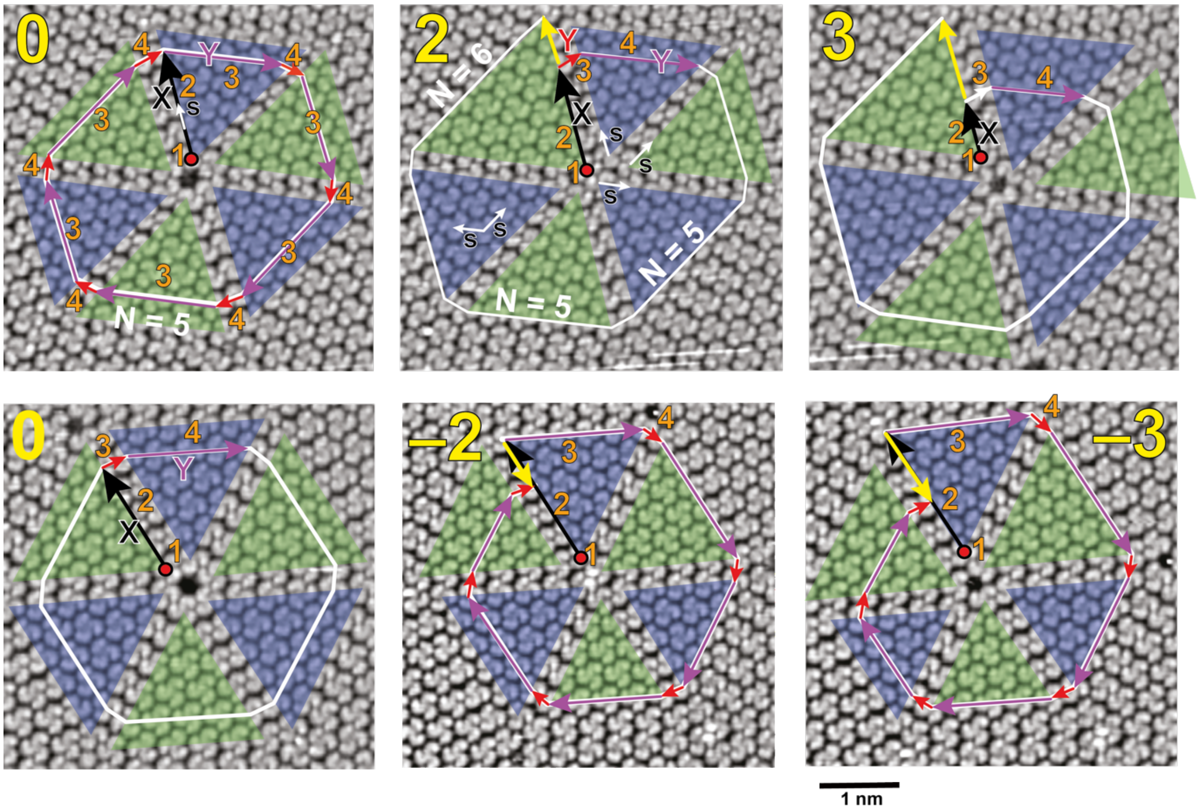

Supplementary Fig. 3 | Topology of nodes. In order to determine the topology of the three different observed nodes the following procedure is applied: As each node is the result of the arrangement of six triangles with hexagonally ordered molecules, the inner tip of one of the triangles serves as starting point (red dot, 1). From there draw a vector **X** along the edge of the triangle of several molecular unit vectors **s** (2). The length of **X** has to be between the triangle size *N–1* and *N–3* (**s** × *N–1* ≥ **X** ≥ **s** × *N–3*; *N* = triangle size in units of **s**). All following operations are performed clockwise. From there draw a vector **Y** across the triangle to the opposite border with same distance to the inner triangle tip (3). Move to adjacent triangle border molecule that is closest (interdigitated) to last molecule (4). Repeat steps 3 and 4 until crossing a vector pointing at the same direction as **X**. If **X** was chosen on the right side of a triangle, order of steps 3 and 4 need to be invers (i.e. crossing to adjacent triangle border before moving across triangle). The topology is now defined by the distance in molecular unit vectors **s** of the endpoint of the operation to the tip of **X** (no matter if the endpoint is closer or farther away from the inner triangle tip). Nodes in a domain with the *(P)*-enantiomer as majority can have topologies of 0, 2 and 3; for nodes in domains with the *(M)*-enantiomer as majority topologies of 0, –2 and –3 are the result. Scale bar applies to all panels.

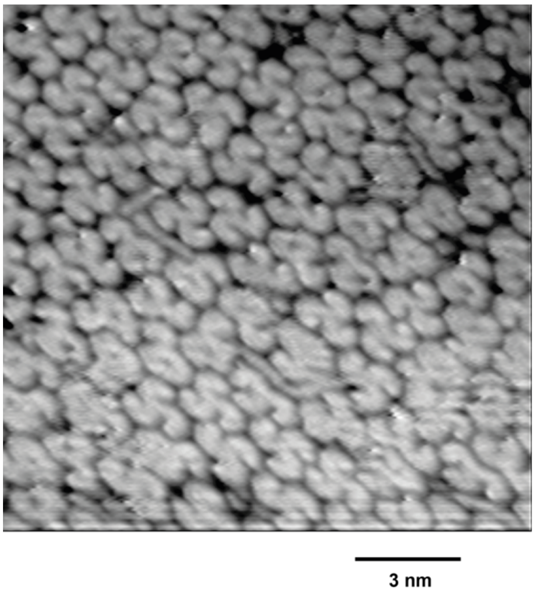

Supplementary Fig. 4 | Cold sample deposition. STM image (15 nm × 15 nm) after deposition at 170 K and cooling to 130 K. Even at high coverage there is no order. Not all molecules are present in their *(M,M,M)*- and *(P,P,P)*-configurations but also as diastereomers with mixed handedness, e.g. as *(M,P,P)*- or as *(P,M,M)*-isomers, appearing therefore in y-shapes.

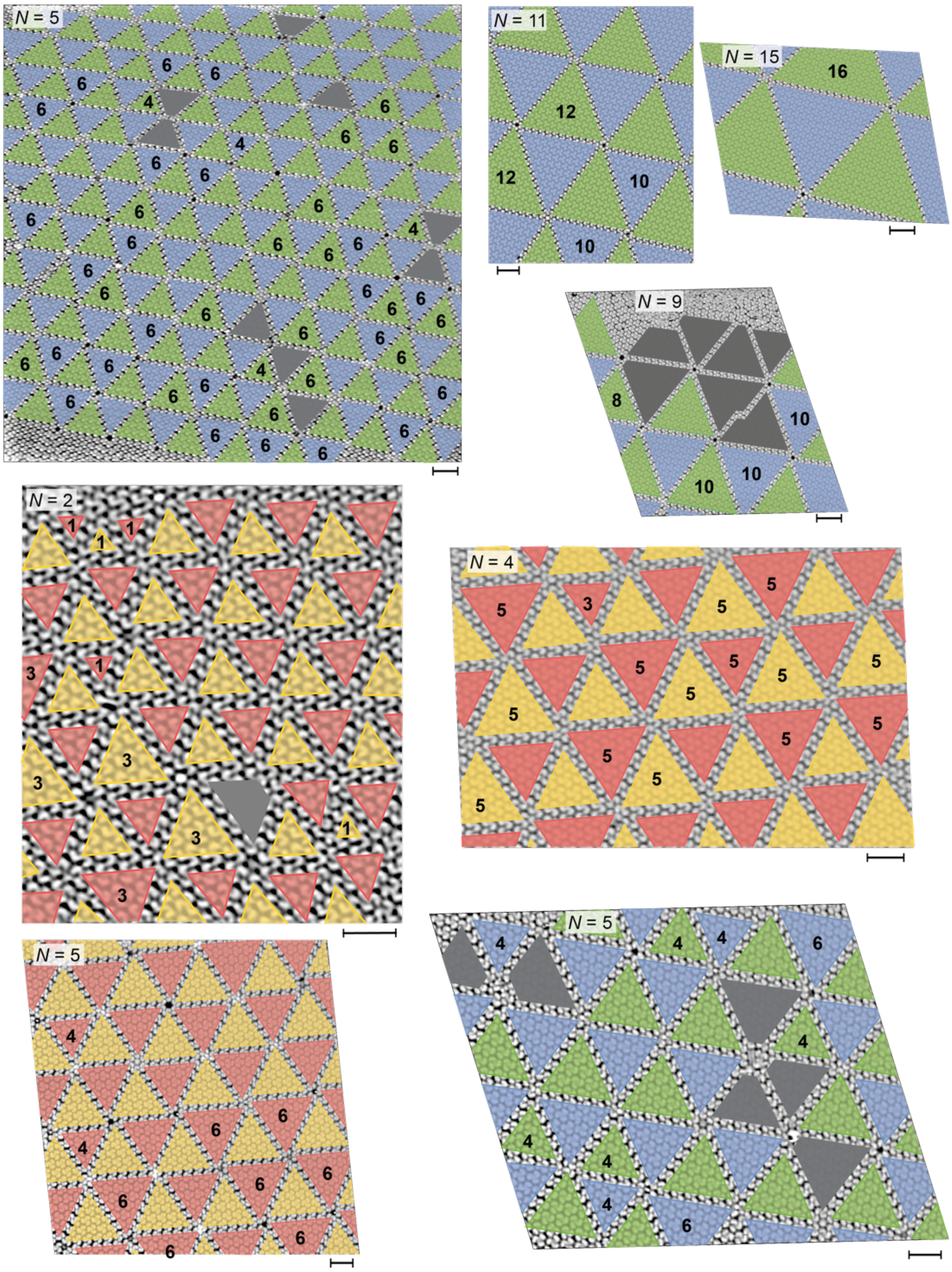

Supplementary Fig. 5 | *N*±1 triangular phases. Examples of STM images acquired from different *N*±1 triangular phases obtained after different identical sample preparations. Semitransparent triangles indicate corresponding color for molecular azimuthal orientation and majority. All STM images were drift corrected, causing the deviation from rectangular shape. The dominating *N* is indicated, while examples of the subordinate-sized ±1 triangles are directly labeled. Incomplete triangles are labeled in grey. Scale bars below the images mark 5 nm.

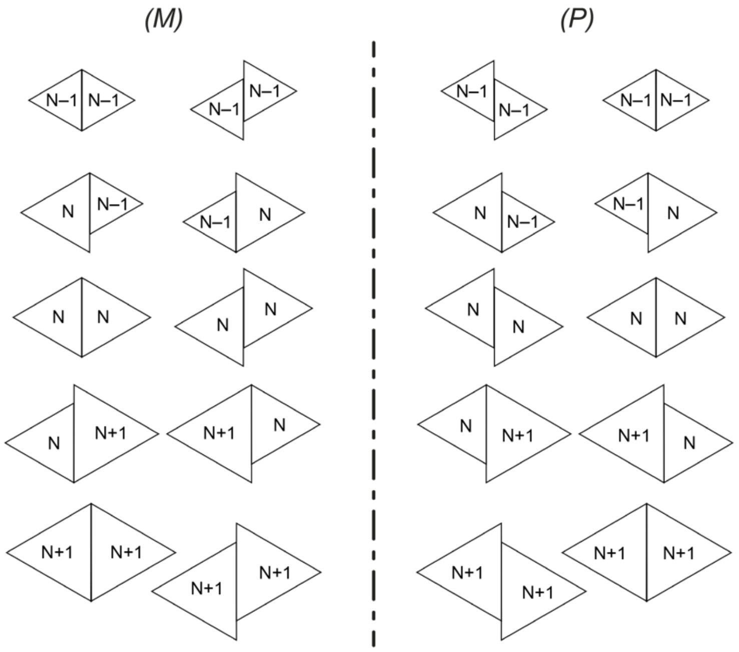

Supplementary Fig. 6 | Combinations of triangle arrangements in *N*±1 topological assembly. 10 possible combinations are identified for each enantiomer. The enantiomer defines the direction of the offset between triangles.

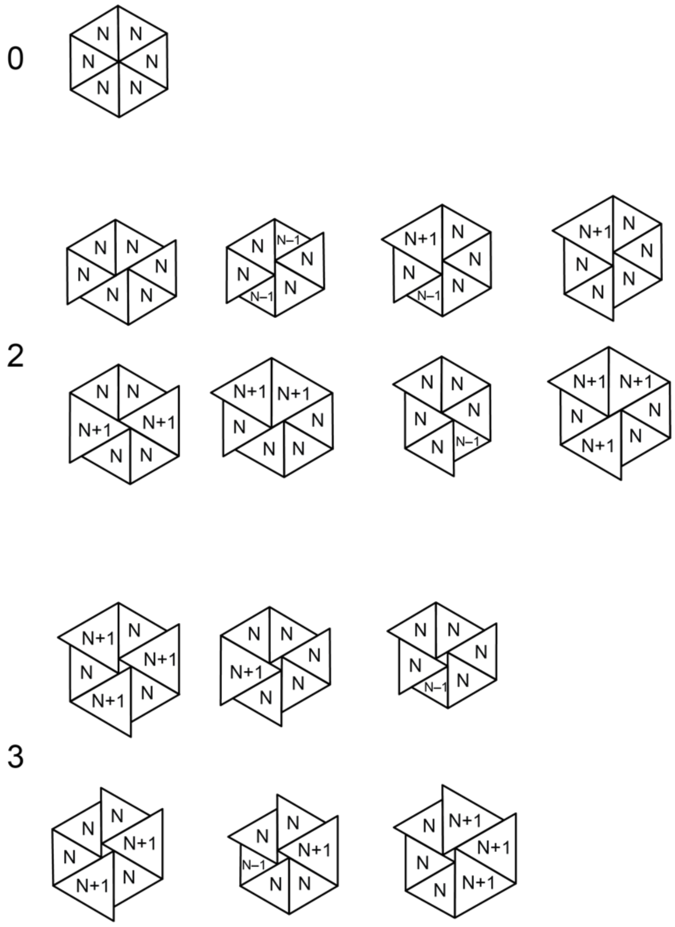

Supplementary Fig. 7 | Observed node types for triangular *N*±1 topological assembly. More node types are theoretically possible but have not been identified experimentally.

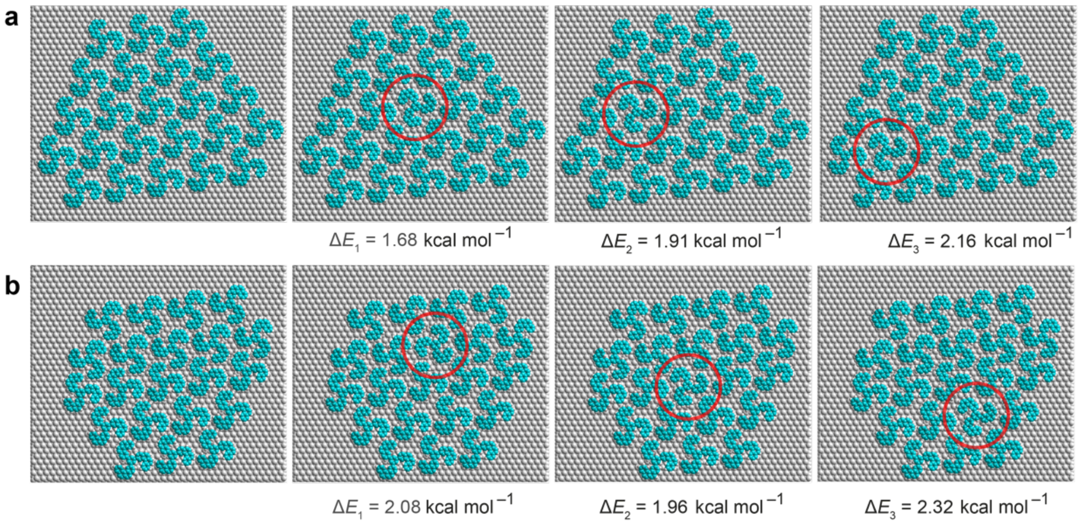

Supplementary Fig. 8 | Molecular mechanics of inclusion of a wrong enantiomer. The inclusion of a molecule with opposite handedness induces an energy penalty of only around 2 kcal mol^{-1}. The position within a given ensemble leads only to a small variation of this value.

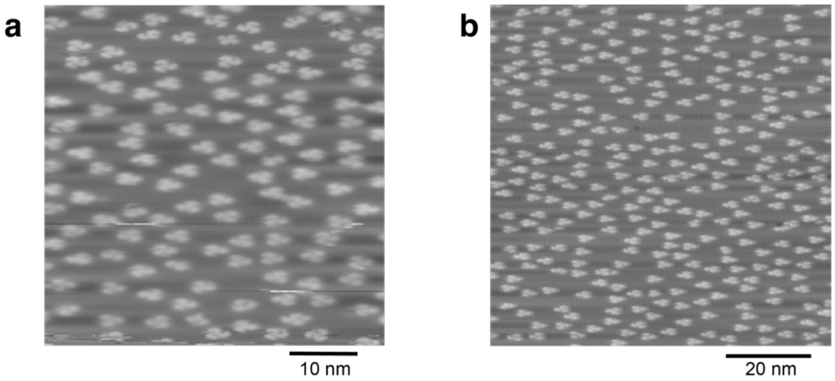

Supplementary Fig. 9 | Low coverage STM investigation. a STM image (T = 10 K) at low coverage after deposition at room temperature. Cooling rate: ≈ 0.22 K s^{-1}. **b** STM image (T = 10 K) at low coverage after deposition at room temperature. Cooling rate: ≈ 0.22 K s^{-1}. The absence of aggregation into islands suggests very small attractive interactions and dipolar repulsion due to charge transfer or Pauli repulsion caused by interaction with the substrate. Hence, molecules interact basically only close to full monolayer coverage.

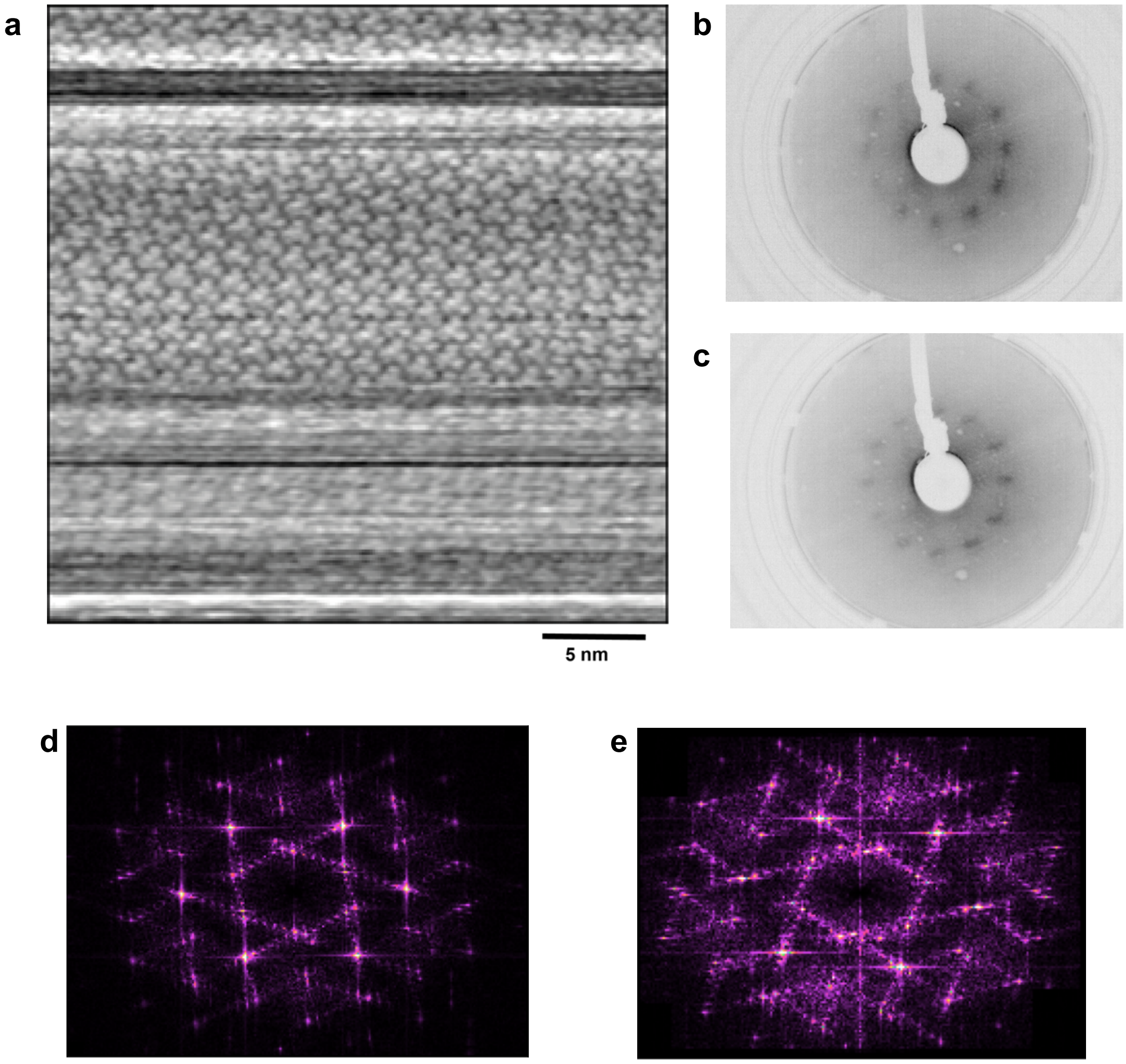

Supplementary Fig. 10 | Order at high temperature. a STM image of a closed-packed layer at room temperature. **b** Low-energy electron diffraction (LEED, E = 43 eV) pattern of a monolayer at 40 °C. **c** LEED pattern (E = 43 eV) of a monolayer at 80 °C. **d** Inverse Fourier transformation of STM image of the triangular phase containing excess of *(P)*-t[4]HB. **e** Inverse Fourier transformation of STM image of the triangular phase containing excess of *(M)*-t[4]HB.

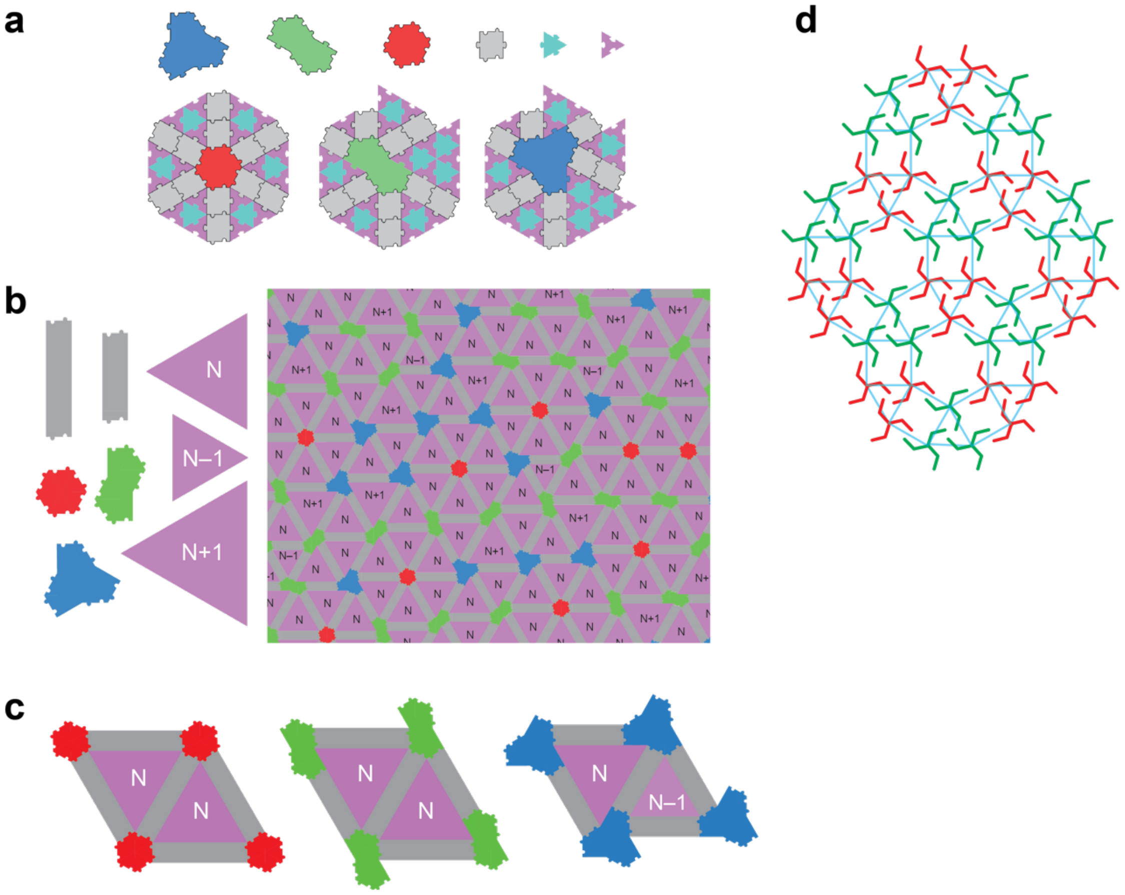

Supplementary Fig. 11 | Set of tiles mimicking the observed self-assembly. a Set of tiles involving bulges and notches that guarantee interlocking compatible with the molecular self-assembly. Magenta and cyan tiles represent the triangular sub-domains that form triangles of arbitrary large size. Triangle boundaries are represented by grey tiles. Their chirality is reflected by the mirror-symmetry breaking position of bulges. **b** Set of tiles using the N±1 triangle size topology and example of tiling with this tile set. A larger example of tiling is presented in Supplementary Figure 12. **c** Examples of periodic solutions when only node-pure arrangements are considered. **d** The arrangement around a 0-node with triangle size N=1 constitutes a rhombitrihexagonal tiling but is not realistic due to a small covered/uncovered area ratio.

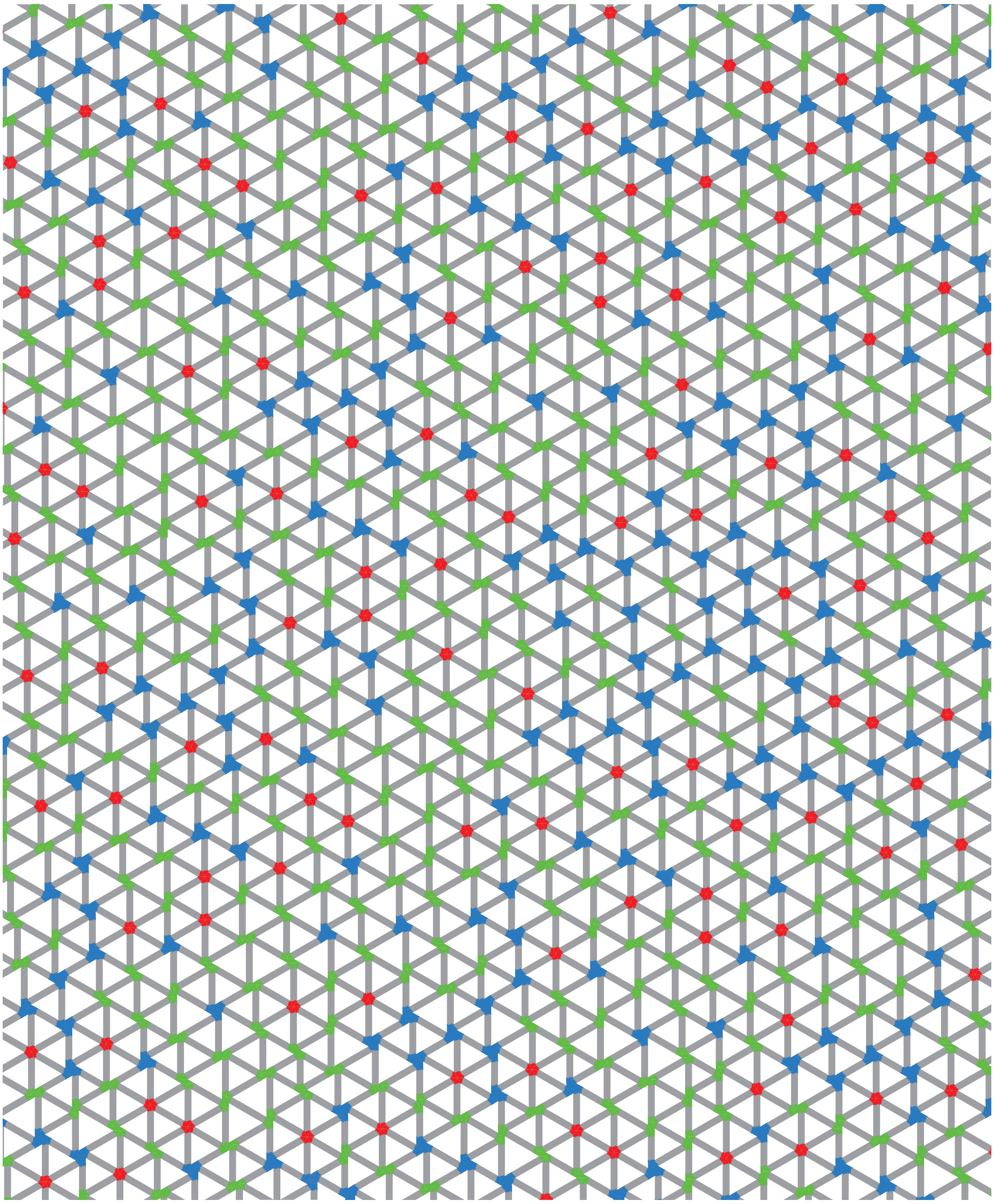

Supplementary Fig. 12 | Topological jigsaw tiling game. Example of an aperiodic tiling using the tile set introduced in Suppl. Fig. 11 based on the N±1 triangle size topology (triangle colors are kept in white).

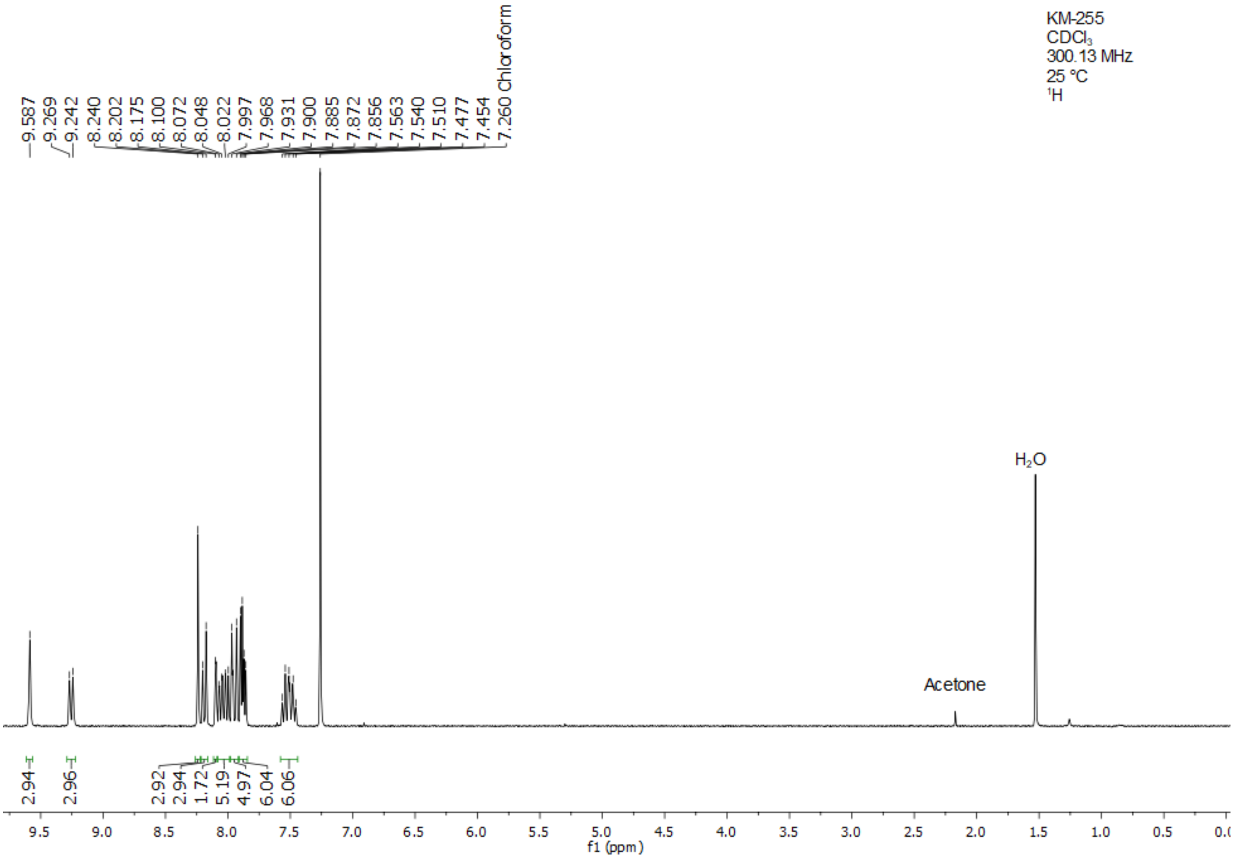

Supplementary Fig. 13 | 1H NMR of t[4]HB.

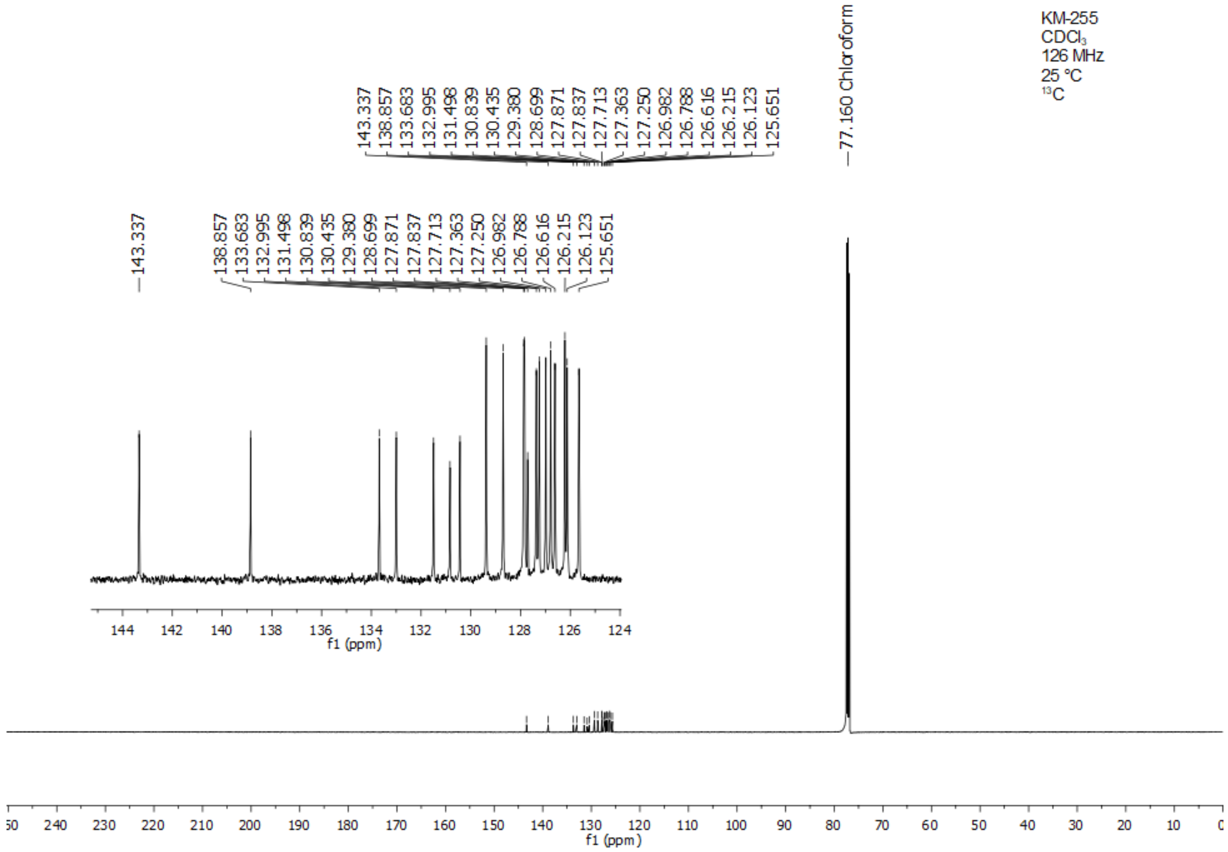

Supplementary Fig. 14 | 13C NMR of t[4]HB.

a

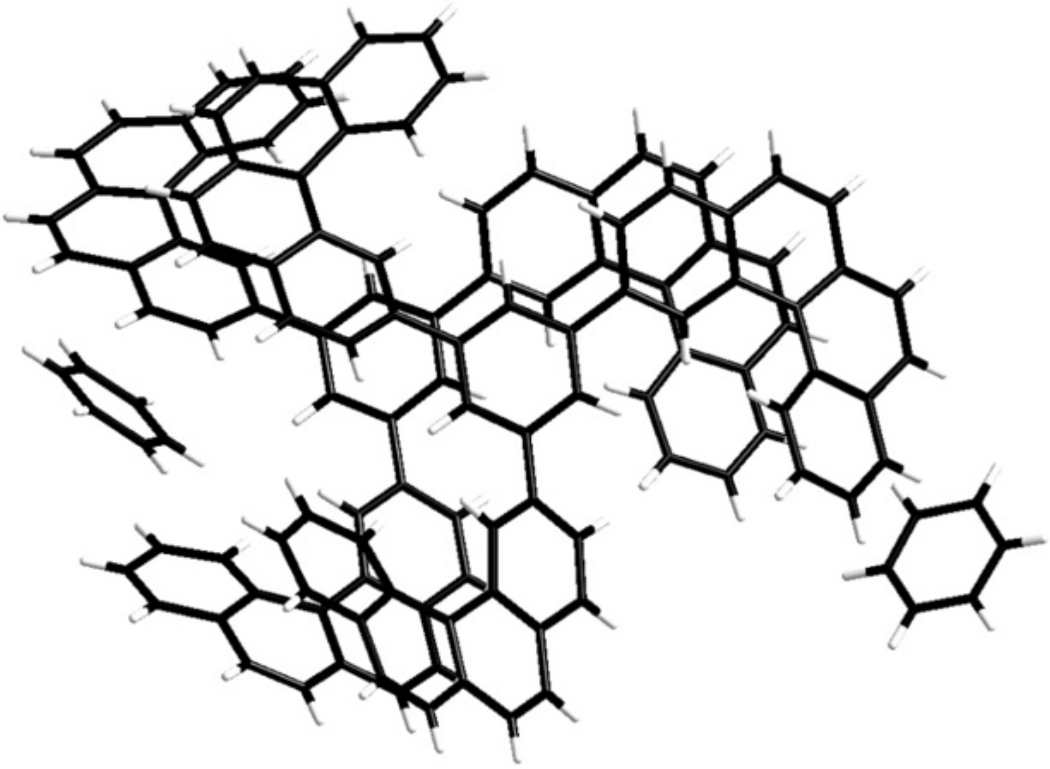

b

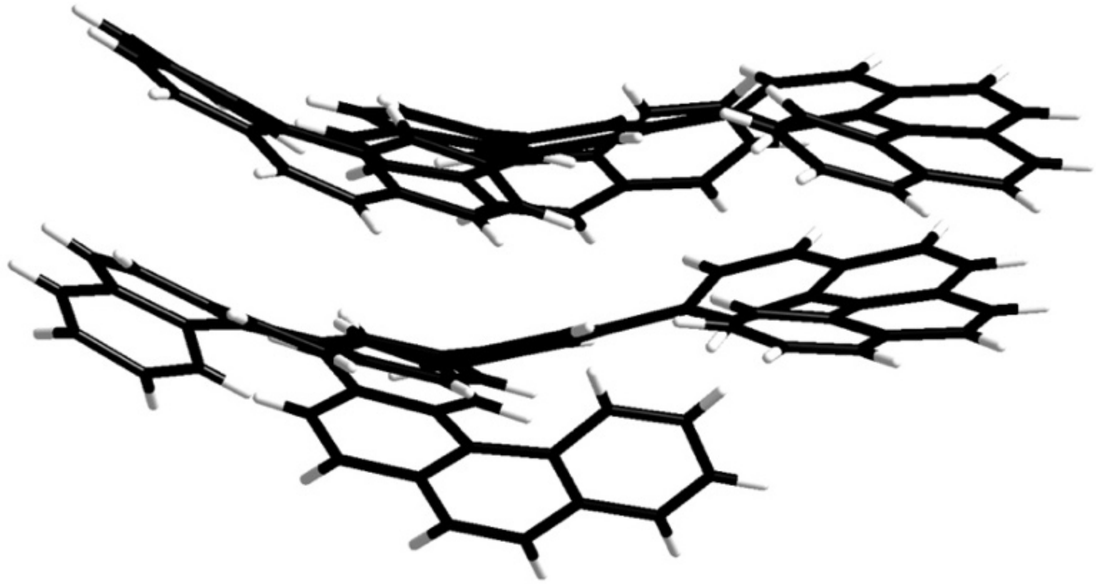

Supplementary Fig. 15 | Molecular Structure of t[4]HB. a Top view of the molecular structure of t[4]HB with benzene molecules in the crystal. **b** Lateral view in which the benzene molecules have been omitted.

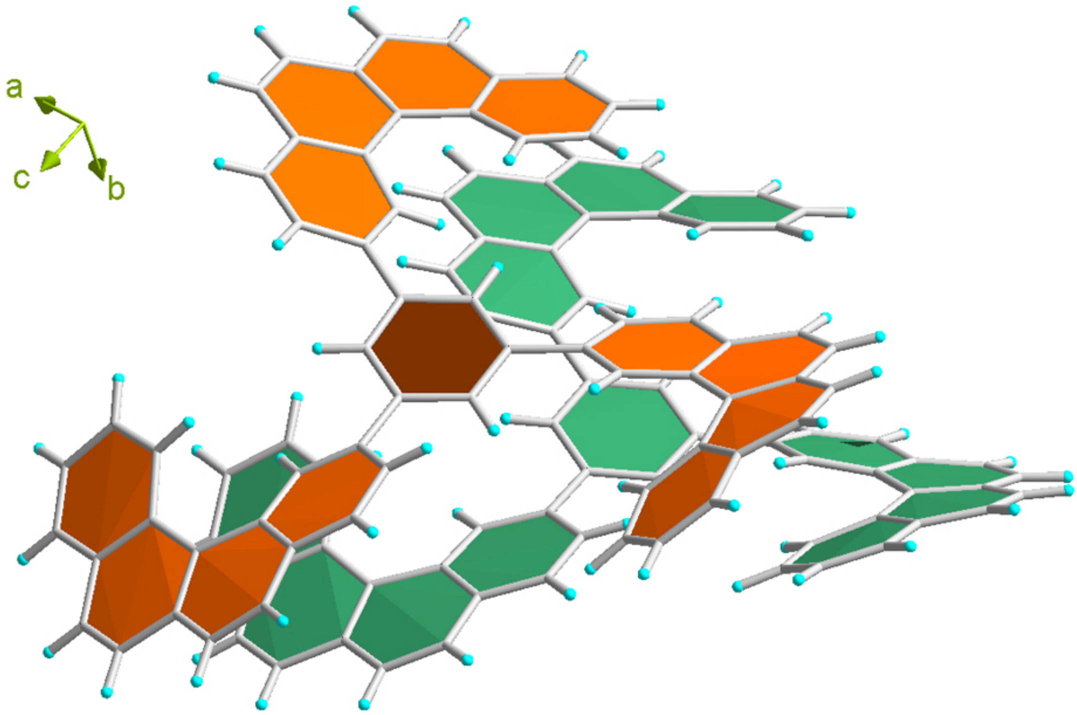

Supplementary Fig. 16 | Crystal structure of t[4]HB. View of the two independent tris(helicene) molecules in the crystal.

Supplementary Table 1 | STM parameters of all presented STM images.

Figure	U (V)	I (pA)
Fig. 1 b	0.30	200
Fig. 1 c top row	0.30	200
Fig. 1 c bottom row	0.30	190
Fig. 1 e	0.30	200
Fig. 1 f	0.83	130
Fig. 1 f insets	0.30	200
Fig. 2 d top row	0.30	200
Fig. 2 d bottom row	0.30	190
Fig. 3 a	0.78	560
Fig. 4 a	0.30	200
Fig. 4 b left	0.96	360
Fig. 4 b right	0.85	210
Supplementary Fig. 1	0.30	200
Supplementary Fig. 2	0.30	200
Supplementary Fig. 4	0.594	250
Supplementary Fig. 5 (N=5, top)	0.25	260
Supplementary Fig. 5 (N=11)	0.83	130
Supplementary Fig. 5 (N=15)	0.42	540
Supplementary Fig. 5 (N=9)	0.42	620
Supplementary Fig. 5 (N=2)	0.50	150
Supplementary Fig. 5 (N=4)	0.39	120
Supplementary Fig. 5 (N=5, left)	0.30	200
Supplementary Fig. 5 (N=5, right)	0.30	190
Supplementary Fig. 9 a	0.48	500
Supplementary Fig. 9 b	1.12	360
Supplementary Fig. 10 a	1.25	60

Supplementary Table 2 | Crystal Data and Structure Refinement for t[4]HB.

Formula	$C_{129}H_{81}$
Molecular Weight	1630.93
Temperature (K)	150.0(1)
Wavelength (Å)	1.54184
Crystal system	Monoclinic
Space group	$P2_1/c$
a (Å)	25.014(2)
b (Å)	7.5767(5)
c (Å)	44.880(4)
α (°)	90
β (°)	103.338(9)
γ (°)	90
V (Å^3)	8276.4(12)
Z	4
Crystal color	Colourless
Crystal size (mm^3)	0.3 x 0.2 x 0.1 mm
D_c (g cm^{-3})	1.309
F(000)	3420
μ (mm^{-1})	0.563
Transmission (min/max)	0.65353/1.00000
θ (min/max) (°)	2.386/76.2
Data collected	34412
Data unique	16780
Data observed	8676
R (int)	0.0829
Nb of parameters	1162
Nb of restraints	0
R_1 [I > 2σ(I)]	0.0827
wR_2 [I > 2σ(I)]	0.1963
R_1 [all data]	0.1531
wR_2 [all data]	0.2624
GOF	1.018
CCDC number	2092008

$$W_{\mathrm{chiral}} = \binom{N_{\mathrm{mol}}}{N_{\mathrm{min}}} = \frac{N_{\mathrm{mol}}!}{(N_{\mathrm{mol}}-N_{\mathrm{min}})!\,N_{\mathrm{min}}!} = \frac{N_{\mathrm{mol}}!}{\mathrm{N}_{\mathrm{maj}}!\,N_{\mathrm{min}}!}$$

Supplementary Equation 1 | Probability of including 'wrong' handedness. Supplementary Eq. 1 gives the total number of possible arrangements for an ensemble in a solid solution of chiral molecules. It is assumed that the distribution of the molecules of opposite handedness (minorities) is random within every arrangement (independent of the surrounding node types and triangle sizes). N_{min}, N_{maj} and N_{mol} represent the numbers of minority molecules, majority molecules and total number of molecules ($N_{\mathrm{mol}} = N_{\mathrm{maj}} + N_{\mathrm{min}}$), respectively.
